# Development of male-larger sexual size dimorphism in a lizard: *IGF1* peak long after sexual maturity overlaps with pronounced growth in males

**DOI:** 10.3389/fphys.2022.917460

**Published:** 2022-08-10

**Authors:** Brandon Meter, Lukáš Kratochvíl, Lukáš Kubička, Zuzana Starostová

**Affiliations:** ^1^ Department of Zoology, Faculty of Science, Charles University in Prague, Prague, Czechia; ^2^ Department of Ecology, Faculty of Science, Charles University in Prague, Prague, Czechia

**Keywords:** body size, bone, growth, hormones, IGF1, reptiles, sexual size dimorphism, testosterone

## Abstract

Squamate reptiles have been considered to be indeterminate growers for a long time. However, recent studies demonstrate that bone prolongation is stopped in many lizards by the closure of bone growth plates. This shift in the paradigm of lizard growth has important consequences for questions concerning the proximate causes of sexual size dimorphism. The traditional model of highly plastic and indeterminate growth would correspond more to a long-term action of a sex-specific growth regulator. On the other hand, determinate growth would be more consistent with a regulator acting in a sex-specific manner on the activity of bone growth plates operating during the phase when a dimorphism in size develops. We followed the growth of males and females of the male-larger Madagascar ground gecko (*Paroedura picta*) and monitored the activity of bone growth plates, gonad size, levels of steroids, expression of their receptors (*AR, ESR1*), and expression of genes from the insulin-like growth factor network (*IGF1*, *IGF2*, *IGF1R,* and *IGF2R*) in livers. Specifically, we measured gene expression before the onset of dimorphic growth, at the time when males have more active bone growth plates and sexual size dimorphism was clearly visible, and after a period of pronounced growth in both sexes. We found a significant spike in the expression of *IGF1* in males around the time when dimorphism develops. This overexpression in males comes long after an increase in circulating testosterone levels and sexual maturation in males, and it might be suppressed by ovarian hormones in females. The results suggest that sexual size dimorphism in male-larger lizards can be caused by a positive effect of high levels of *IGF1* on bone growth. The peak in *IGF1* resembles the situation during the pubertal growth spurt in humans, but in lizards, it seems to be sex-specific and disconnected from sexual maturation.

## Introduction

Animals differ in growth patterns. Some animals stop growing before sexual maturation, e.g. most insects which are not able to change their structural size as imagoes due to rigid exoskeleton, while others used to be assigned as indeterminate growers. Among vertebrates, endotherms (mammals and birds) are traditionally viewed as determinate growers, while non-avian reptiles, amphibians, and fish as indeterminate growers (Kozłowski, 1996; Charnov et al., 2001; Geiger et al., 2014; Hariharan et al., 2015). However, this dichotomy is shaking now. Many reptiles, particularly lizards, do in fact stop growing and attain a final body size. However, they typically do so long after sexual maturation (Frýdlová et al., 2019; Frýdlová et al., 2020). The reasons for this are still unclear, in a previous review we speculated that due to a much lower metabolic rate, growth in ectotherms is slow and that they attain final body size much later, which forces them to start reproduction much before reaching the final, possibly optimal body size. Endotherms with more rapid growth can postpone reproduction after they attain an optimal body size (Meter et al., 2020). Alternatively, it could be explained by the energetically demanding nature of endothermy and a trade-off between reproduction, growth, and body temperature maintenance (Werner et al., 2018). The new perspective on lizards as determinate growers has important consequences for the search for the proximate mechanisms of sexual size dimorphism (SSD), i.e., the size difference between sexes of a species, and its measurement. Under determinate growth, SSD should be best expressed as a size difference between fully grown males and females, and it should reflect the processes largely influencing the cessation of growth in a sex-specific manner.

SSD is widespread in reptiles and can differ in direction even among closely related species (Cox et al., 2007; Starostová et al., 2010). Finding a proximate mechanism of SSD development in vertebrates is a complex task. Several hypotheses on the proximate cause of SSD have been proposed in squamate reptiles. In most squamates as well as in many other vertebrates, there is little if any SSD at hatching, and the early growth is monomorphic (Badyaev, 2002). SSD develops only later in ontogeny through dissociation of male and female growth trajectories. But which sex-specific growth modifiers do in fact drive this dissociation? Sexes in some vertebrates do not differ in genomes, e.g. in sequential hermaphrodites and species with environmental sex determination (Janzen and Phillips, 2006; Johnson Pokorná and Kratochvíl, 2016; Todd et al., 2016; Katona et al., 2021), but still often substantially differ in body size. In them, sex-specific growth modifiers cannot be attributed to genetic differences between sexes. But also, in species with genotypic sex determination, an overwhelming majority of tetrapods (Kostmann et al., 2021), sex-specific modification of growth is mostly triggered by differential expression during development, not directly by sex-linked genes. It can be exemplified by how easily growth can be altered by gonad manipulations, e.g. ovariectomy leads to male-typical growth in several squamate species (Cox, 2006; Starostová et al., 2013; Cox et al., 2016; Kubička et al., 2017; Cox, 2019).

The cost of reproduction hypothesis postulates that SSD is plastic with respect to energy allocation. SSD should reflect energetic costs of reproduction and the sex allocating less to reproduction should grow larger (Cox, 2006; Cox et al., 2009; Hayward and Gillooly, 2011; Cox et al., 2016). This hypothesis was supported by manipulative experiments in male-larger species, where ovariectomized females were masculinized in growth (Cox, 2006; Cox and Calsbeek, 2010; Starostová et al., 2013; Cox et al., 2016). Nevertheless, unilateral ovariectomy, dramatically decreasing the allocation to reproduction, but at the same time preserving the cycling of ovarian hormones, did not lead to enhanced structural growth in male-larger gecko *Paroedura picta*. It suggests that ovariectomy does not remove only the energetic costs of reproduction, but also endocrinologically active organs producing hormones affecting growth (Kubička et al., 2017).

The ontogeny of SSD was also suggested to be controlled by circulating levels of male gonadal androgens, which should trigger enhanced growth in male-larger species and inhibit growth in female-larger species (Cox et al., 2005; Cox et al., 2009; Duncan et al., 2020). However, most of the support came from relatively short-term studies done under the view that lizards are indeterminate growers, and there was thus no control in these studies on whether the animals already stopped growth or not. The long-term growth experiments comparing growth in castrated and control males do not support the role of male gonadal androgen neither in male-larger species, namely the gecko *P. picta* (Starostová et al., 2013; Kubička et al., 2015) and the chameleon *Chamaeleo calyptratus* (Bauerová et al., 2020), nor in the female-larger gecko *Aeluroscalabotes felinus* (Kubička et al., 2013). It was also noted that the increase of testosterone levels in males does not coincide with the period of sexually dimorphic growth in a rattlesnake and the gecko *P. picta* (Taylor and DeNardo, 2005; Kubička et al., 2022).

An alternative proximate mechanism of SSD development is the control by ovarian hormones (Schmidt et al., 2000; Starostová et al., 2013). This hypothesis was supported by a coincidence in the start of female reproductive cycles with the feminization of growth (Starostová et al., 2013); by a pattern of female-typical growth (Kubička et al., 2022); and by the observation of defeminization of growth after ovariectomy and by the strong negative effect of exogenous estradiol on growth in male-larger gecko *P. picta* (Kubička et al., 2017).

The presented pattern of growth in squamates suggests that the sex-specific growth modifier should not be expressed at the early growth when growth is not sexually dimorphic. It should be detectable at the time of dimorphic growth before the cessation of bone prolongation. It should keep temporarily the higher activity of bone growth plate in the larger sex, and switch again to a non-dimorphic level (or at least it should lose its positive effect on growth in the larger sex) after senescence of growth plates.

To uncover the candidate sex-specific growth modifier, we monitored the growth, the activity of growth plates, size of reproductive organs, and levels of testosterone and estradiol in males and females of *P. picta* from hatching till the age when growth is already stopped, or at least when it is negligible (Kubička et al., 2022). Based on their ontogenetic stage, we selected individual age groups from this experiment, and we measured in them the expression of candidate genes, potential sex-specific regulators in livers, the major metabolic organ. Namely, we measured the expression of receptors of steroid hormones and members of the insulin/insulin-like growth signaling pathway: estrogen receptor alpha (*ESR1*), androgen receptor (*AR*) as well as insulin-like growth factors one and two (*IGF1*/*IGF2*) and their receptors (*IGF1R/IGF2R*).

The insulin and insulin-like signaling molecular networks are composed of many receptors but mainly the insulin-like growth factor network has a strong relation to growth. IGF1 and its counterpart IGF2 have roles in cell growth and proliferation as they are mediators of the growth hormone action (Schwartz and Bronikowski, 2016). The role of IGF1 and IGF2 in the growth of reptiles has been well documented (Reding et al., 2016; Beatty and Schwartz, 2020; Duncan et al., 2020; Marks et al., 2021). IGF1 has been assumed to be a postnatal growth factor and IGF2 to be a prenatal growth factor, but this assumption has been challenged in reptiles (Beatty and Schwartz, 2020). IGF1 and IGF2 compete to bind to insulin receptors and IGF1R which have a role in cell proliferation and growth (Denley et al., 2005; Schwartz and Bronikowski, 2016). IGF1 plasma levels have also been associated with reproduction (Guillette et al., 1996; Sparkman et al., 2010), and its gene expression is affected by nutrition in reptiles (Duncan et al., 2015; Marks et al., 2021). The role of IGF1 in growth in humans and mice is best transcribed in its role in postnatal bone elongation. Hepatic levels of IGF1 have a role in longitudinal bone growth as well as local IGF1 levels in growth plates while affecting chondrocyte differentiation and maturation and therefore their closing (Yakar et al., 2018; Racine and Serrat, 2020). Plasma levels of IGF1 were found to be major determinants of sexually biased skeletal dimorphism in mice and tied with puberty (Callewaert et al., 2010). Radial bone growth has also been found to be affected by IGF1 levels (Yakar et al., 2018).

Previous papers on reptiles using absolute quantification gene expression indicated that IGF2 has a role in postnatal growth in reptiles with levels being higher than in IGF1 (Reding et al., 2016; Beatty and Schwartz, 2020). This is similar to the plasma levels trends in other vertebrates (de Beer et al., 2008; Fowke et al., 2010) but not rodents (Brown et al., 1986). IGF2 binds to IGF2R which acts negatively toward IGF2 since it degrades IGF2 to maintain non-excessive levels and therefore negatively regulates the insulin/insulin-like signaling network (Ghosh et al., 2003; Denley et al., 2005). This function is still unclear in reptiles (Schwartz and Bronikowski, 2016). However, we do know that this binding is potentially significant in lizards (Sivaramakrishna et al., 2009). *IGF2* has also been found to have a highly conserved sequence in squamates (Schwartz and Bronikowski, 2016) while in contrast, *IGF1* is quite variable amongst reptiles (Schwartz and Bronikowski, 2016; Yamagishi et al., 2016).

The androgen pathway and namely the hormone testosterone has been linked to sexual dimorphism in the past (Zauner et al., 2003; Cox et al., 2005, 2007). Testosterone and dihydrotestosterone have been found to trigger their biological activities through binding to the AR (Heinlein and Chang, 2002). Estrogens bind to two different receptors, the ESR1, and estrogen receptor beta (ESR2) which are expressed differently in different tissues and can have differential roles (Sanger et al., 2014; Bondesson et al., 2015).

Our study assesses the gene expression of target genes by quantitative reverse transcription PCR (RT-qPCR) to determine relative gene expression between selected stages of growth to uncover genes responsible for sexually dimorphic growth in a male-larger species. We expect that a member of the crucial pathways modifying growth in a sex-specific manner should be differentially expressed during the time when sexual dimorphism emerges in the ontogeny.

## Materials and methods

### Model species

The gecko *P. picta* has become a model species for studies of reptile growth and ontogeny (reviewed in Meter et al., 2020). The species was used for this experiment specifically due to its male-larger SSD and availability of genetic information with both whole-genome and transcriptome data being available (Hara et al., 2015; Hara et al., 2018), which enabled the design of primers for candidate genes. Our working hypothesis of the male-larger SSD in our model species *P. picta* is the combative and territorial nature of males driving intersexual selection. Such male-typical territorial behavior was confirmed in a series of experiments (e.g., Golinski et al., 2014; Schořálková et al., 2018).

### Experimental animals

**FIGURE 1:**
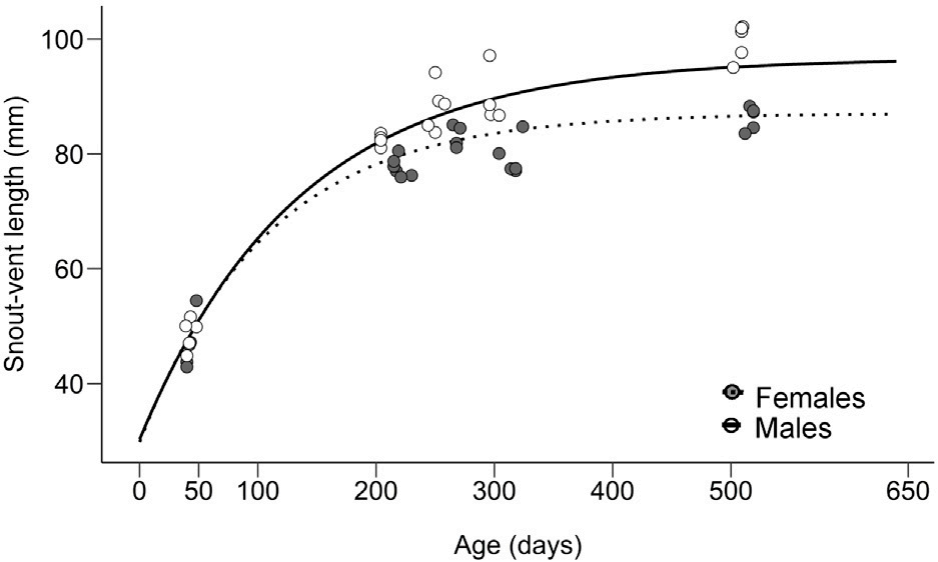
Sex-specific growth curves estimated from 256 individuals (data from Kubička et al., 2022.) and snout-vent lengths in males (solid line, white circles) and females (dotted line, grey circles) of *Paroedura picta* used for gene expression measurements.

For the present study, we used individuals of *P. picta* from the specifically selected age groups that were reared in the experiment conducted by Kubička et al. (2022). Briefly, Kubička et al. (2022) followed growth in 256 individuals of *P. picta*, that were the progeny of the wild-caught animals or of the first generation in captivity. The experimental individuals were incubated and held individually after hatching at the constant temperature of 30°C (±0.2) and 12:12 h light–dark cycle. Animals were kept in a standardized box with a sandy substrate, water dish, and shelter and fed *ad libitum* twice a week with crickets powdered with vitamins and minerals. The growth of experimental animals was followed from hatching to up to the age of *c*. 22 months and divided into 14 age groups with approximately 6-week intervals. Animals were regularly weighed, and their snout-vent length (SVL) was measured. Maintaining typical physiological processes such as hormonal cycles in females was ensured by allowing animals to mate and reproduce. Once a female reached 4 months of age or a body mass of 7 g, it was mated with an unrelated experimental male of the same age. At the time the animals reached the age required for a particular cohort (ranging between 0 and 620 days), they were sacrificed by rapid decapitation, and samples were collected for future analyses. To maintain similar food availability across all age cohorts animals were not starved prior to the decapitation. Based on growth data for all individuals, the onset of sexually dimorphic growth, revealed by the breakpoint analysis, occurred at *c*. 180 days of age (Kubička et al., 2022). For our analyses, we selected animals at the beginning of the growth curve before the onset of dimorphic growth (≈42 days), at the time when males have more active bone growth plates and SSD was clearly visible (≈255 days; with two age groups added for target gene *IGF1* ≈ 211 days; ≈ 307 days) and the end of the growth curve, i.e. after the period of pronounced growth in both sexes (≈512 days) (see Figure 1). Final body mass and SVL were taken immediately before euthanasia. The sex of each individual was confirmed by gonadal inspection and the mass of testicles and ovaries was recorded (except for the ovaries of 42 days old females as they were too small). Plasma for all 256 animals was used for measurements of levels of estradiol and testosterone, as described in Kubička et al. (2022) and showed in Supplementary Figures S1, S2, which gave us information about hormonal profiles across the ontogeny. Due to the necessity of pooling some plasma samples in small individuals, we did not use hormone levels directly in our statistical analyses. All raw data used in Kubička et al. (2022) are available in the Mendeley database (doi:10.17632/fxcdd6j4sh.1).

### Sample collection, RNA isolation and cDNA synthesis

Liver samples were collected from individuals of both sexes and different age groups. The liver was chosen as the target tissue because estrogen and testosterone metabolize in the organ while gonads were too small. Furthermore, the liver was also chosen as it had a higher expression of *IGF1* and *IGF2* than other tissues in several reptiles (Reding et al., 2016; Duncan et al., 2020; Marks et al., 2021), and differences in the expression of *IGF1R/IGF2R* in the liver were found between fast versus slow-growing sexually dimorphic snake (Reding et al., 2016). After dissection, the liver samples were quickly cut into small pieces and stored in RNA*later* (Sigma-Aldrich Inc., Missouri, United States) at −20°C until used for RNA extraction. RNA was extracted according to the manufacturer’s protocol using the HighPure RNA tissue kit (Roche, Germany) and treated with DNase with a standardized amount of about 10 mg of liver tissue for each extraction. Each RNA sample had its concentration measured using the Quantus fluorometer (Promega Corporation, Madison, United States), and the Agilent 2,100 Bioanalyzer and Agilent RNA 6000 Nano Kit (Agilent Technologies, California, United States), were used to assess RNA integrity. Usually, samples with RNA integrity number (RIN) under 3 are considered heavily degraded, and samples with RIN between 3 and 5 are partially degraded (Farrell 2017). Our samples had RIN ranging from 4.6 to 9.8 and only a single sample had a RIN below 5 (4.6). The purity of RNA measured as *A*260/*A*280 ratios was determined with the NanoDrop One (Thermo Fisher Scientific Inc., Massachusetts, United States) and only samples with acceptable ratios (between 1.9 and 2.15) were kept in this experiment, as ratios around 2 are considered pure RNA (Farrell, 2017). Once extracted RNA samples were divided into aliquots and diluted to 50 ng/μl for the cDNA synthesis. All RNA samples were stored at −80°C. The RNA samples were reverse transcribed using the TATAA GrandScript cDNA synthesis kit (TATAA Biocenter, Sweden) with a standard amount of 500 ng of total RNA in each sample.

### Quantitative reverse transcription PCR

Primer pairs for six target genes and three reference genes were designed (Table 1) using the BioEdit software (Hall, 1999) and published transcriptome of our target species (Hara et al., 2015; available at https://transcriptome.cdb.riken.jp/reptiliomix). In addition to the *P. picta* transcriptome, mRNA sequences for each gene of interest of different vertebrate species from the GenBank database were used in primer design. Reference gene *18S* primers were the ones previously used in reptiles (Plot et al., 2012; Rollings et al., 2017). Products of each primer pair were sequenced and aligned with other mRNA sequences to verify their authenticity. The efficiency of each primer pair (Table 1) was assessed by creating a standard curve and using the software of the Lightcycler 480 PCR machine (Roche, Germany) for calculation. A pooled cDNA of samples from the different age and sex groups was used to create standard curves in dilution series 100,000; 20,000; 4,000; 800; and 160 copies to assess primer efficiency. The thermal cycle used for all the assays was as follows: 95°C for 30 s of activation, then 40 cycles of 5 s at 95°C followed by 58°C for 15 s and 72°C for 10 s of amplification cycles. Finally, a melting curve of 60–95°C was done after the final cycle to assess for possible secondary products.

**TABLE 1:** Primer pairs of target and reference genes.

Gene	Forward 5′-3′	Reverse 5′-3′	Amplicon size (bp)	Efficiency (%)
Target				
* IGF1*	*GTG​GAG​CTG​AGC​TGG​TTG​AT*	*ACA​GCT​CTG​GAA​GCA​GCA​TT*	140	95
* IGF1R*	*GTG​CTG​GAC​TTC​CAA​CCA​CT*	*GGT​GGC​AGC​ACT​CAT​TTT​G*	86	88.4
* IGF2*	*TGT​GGT​TGA​TTC​TGC​CTC​TG*	*TTGAGCCGGCCTCTGTTT*	138	85.8
* IGF2R*	*GCG​ACC​TCG​TTT​GGT​AAC​AT*	*GGG​AAG​ACA​CAA​AGC​TCA​TCC*	131	94.2
* ESR1*	*TGC​TGA​CAG​AGA​GCT​GGT​ACA*	*TCT​AGC​CAA​GCA​CAT​TCC​AG*	108	94.9
* AR*	*TGG​AAG​CTG​CAA​GGT​CTT​TT*	*GCA​TCT​TCA​GAT​TGC​CCA​GT*	190	90.5
Reference				
* GAPDH*	*AGCTGAACGGCAAACTGA*	*GTC​ATA​CTT​GGC​TGG​CTT​GG*	101	84.4
* 18S*	*GAG​GTG​AAA​TTC​TTG​GAC​CGG*	*CGA​ACC​TCC​GAC​TTT​CGT​TCT*	92	93.4
* YWHAZ*	*TGA​TGT​GCT​GTC​TCT​TTT​GG*	*TTG​TCA​TCA​CCA​GCA​GCA​A*	129	81.6
* HRPT1*	*TGA​CAA​GTC​CAT​TCC​CAT​GA*	*TTC​CCA​GTT​AGG​GTC​GAG​AG*	117	91

All primers, except the primer pair for 18S gene (Plot et al., 2012; Rollings et al., 2017) were designed directly for *P. picta*.

For the gene expression measurement, Cq values from 5 samples of each age/sex group were measured in triplicates in each assay (or primer pair). For each assay, triplicates of no-template controls (wells with nuclease-free water instead of cDNA—NTC) were used to assess for contaminations in reagents. In each well was used 5 μl of the TATAA® SYBR® green master mix (TATAA Biocenter, Sweden), 2.6 μl of nuclease-free water, 0.4 μl of forward and reverse primer (10 μM) respectively of the assay and finally 2 μl of the sample cDNA for a total of 10 μl. To ensure that comparisons could be drawn with all the reference and target assays, an interpolate calibrator (TATAA IPC kit, TATAA Biocenter, Sweden) was used on each plate in replicates on the same wells.

The relative gene expression of each individual for each target gene was calculated from the raw Cq values using the program R and the interface R studio (RStudio Team, 2019; Core Team, 2020). We first calculated mean Cq from technical replicates for each individual and target and reference gene. The R script used from our design followed a combination of the Vandesompele and Pfaffl method which considers the efficiency (Pfaffl, 2001) of all targets and the multiple reference genes (Vandesompele et al., 2002). The script also allowed for interplate calibration. This script for data analysis and the figures presented were done using R and several packages including the package “outliers” for statistical analysis, and the “ggplot2” package for a graphical representation (Komsta, 2011; Wickham, 2016). A number of packages were also used for data processing: “Tydiverse”, “dplyr”, “psych”, “stringr”, and “writexl” (Wickham, 2019; Wickham et al., 2019; Sievert, 2020; Ooms, 2021; Revelle, 2021; Wickham et al., 2021). Biological outliers of the groups for each target gene were signaled using statistical tests while triplicates were selected in accordance with their coefficient of variation. Triplicate outliers were verified from the raw Cq values while biological outliers were identified using the final relative expression.

For all reference genes and target genes, the efficiency of the primers (Table 1) was considered before normalization and calibration. Considering the efficiency of each gene allows for a more accurate calculation of the relative expression as described by Pfaffl (2001). For normalization the use of several reference genes opted for a more accurate normalization as described by Vandesompele et al. (2002), especially considering the species of interest is not commonly used for gene expression studies and finding a single adequate reference gene would be difficult. We selected four reference genes based on previous experimental work. Glyceraldehyde 3-phosphate dehydrogenase (*GAPDH*) has been used in mammals and reptiles (Coulson et al., 2008; Sanger et al., 2014) with limits under caloric restrictions (Gong et al., 2016; Marks et al., 2021). The 18S ribosomal RNA genes (*18S*) had a primer pair already validated previously in reptiles (Plot et al., 2012; Rollings et al., 2017). Gene tyrosine 3-monooxygenase/tryptophan 5-monooxygenase activation protein zeta (*YWHAZ*) had previously been used as a reference gene in several RT-qPCR studies in different tissue types (Coulson et al., 2008; Al-Sabah et al., 2016). Finally, the gene hypoxanthine-guanine phosphoribosyl transferase (*HRPT1*) was also found to be a suitable candidate in different species for different tissues (Stassen et al., 2015; Wang et al., 2018). A geometric mean of values of all four reference genes was taken as a reference for the measurement of target gene expression (Vandesompele et al., 2002).

Subsequent to the normalization step a group was chosen as a calibrator (females of age group 42 days) to calculate each sample’s relative expression to the mean of that group as described in the established methods (Livak and Schmittgen, 2001; Pfaffl, 2001). Finally, the resulting calculation was log-transformed (natural logarithm transformation) to give the final relative gene expression of each individual for each target gene as described in our Supplementary Material (Supplementary Table S1).

### Statistical analysis

All statistical tests were done with the R program and the interface R studio (RStudio Team, 2019; Core Team, 2020). Biological outliers for each age and sex group were assessed using both Dixon and Grubbs’ tests using the package “outliers’ (Komsta, 2011). For triplicates coefficients of variation above 30% were deemed unacceptable and the replicates were either redone or two of the three Cq-values in a triplicate were kept if one was a clear outlier. Shapiro-Wilk test was used to check for normal distribution in the relative gene expression. Statistically significant differences between groups were assessed using ANOVA tests with sex/age groups as a factor followed by *post hoc* Tukey tests. The relationship between the natural logarithm (ln) transformed mean mass of testicles and ln-transformed *IGF1* expression or ln-transformed body mass was analyzed using Spearman’s correlation coefficient. For all statistical tests, the significance threshold was set at *p* = 0.05.

## Results

The growth curves of *P. picta* individuals show rapid, non-dimorphic growth at the beginning, then a more pronounced cessation of growth in females, followed by a period of the stagnation of body size in both sexes, with males being in the last two segments considerably larger in body size (Figure 1; Kubička et al., 2022).

Measurements of gene expression identified as biological outliers with both Grubbs’ and Dixon tests were taken out of the gene expression analysis. Altogether, there were only few such excluded measurements: the expression value was excluded for the gene *IGF1* in a 211-day-old male (Grubbs’ test: *p* = 0.041; Dixon test: *p* = 0.036), for *ESR1* in a 255-day-old female (Grubbs’ test: *p* = 0.045; Dixon test: *p* = 0.036) and for *IGF1R* in two 255-day-old females on two separate tests (Grubbs’ test: *p* = 0.041; Dixon test: *p* = 0.031/Grubbs’ test: *p* = 0.024; Dixon test: *p* = 0.025). Finally, *AR* had two outliers, a 42-day-old female (Grubbs’ test: *p* = 0.016; Dixon test: *p* = 0.011) and a 255-day-old male (Grubbs’ test: *p* < 0.0001; Dixon test: *p* = 0.004). In addition, triplicates for the measurements of the expression of the gene *IGF1* were too variable in a 307-day-old male. In total, we excluded 7 out of 350 total measurements (2.0%). The statistics with the inclusion of the outliers would lead to the same interpretation of the results with the exception of *ESR1* which has no significant trend with the presence of its outlier.

Gene expression only differed significantly in genes *IGF1, ESR1,* and *AR* between males and females of age 42, 255, and 512 days. For other genes there was no significant difference using ANOVA tests: *IGF1R* (F_5,22_ = 2.589; *p* = 0.055), *IGF2* (F_5,24_ = 0.748; *p* = 0.596), *IGF2R* (F_5,24_ = 0.448; *p* = 0.811).

**FIGURE 2:**
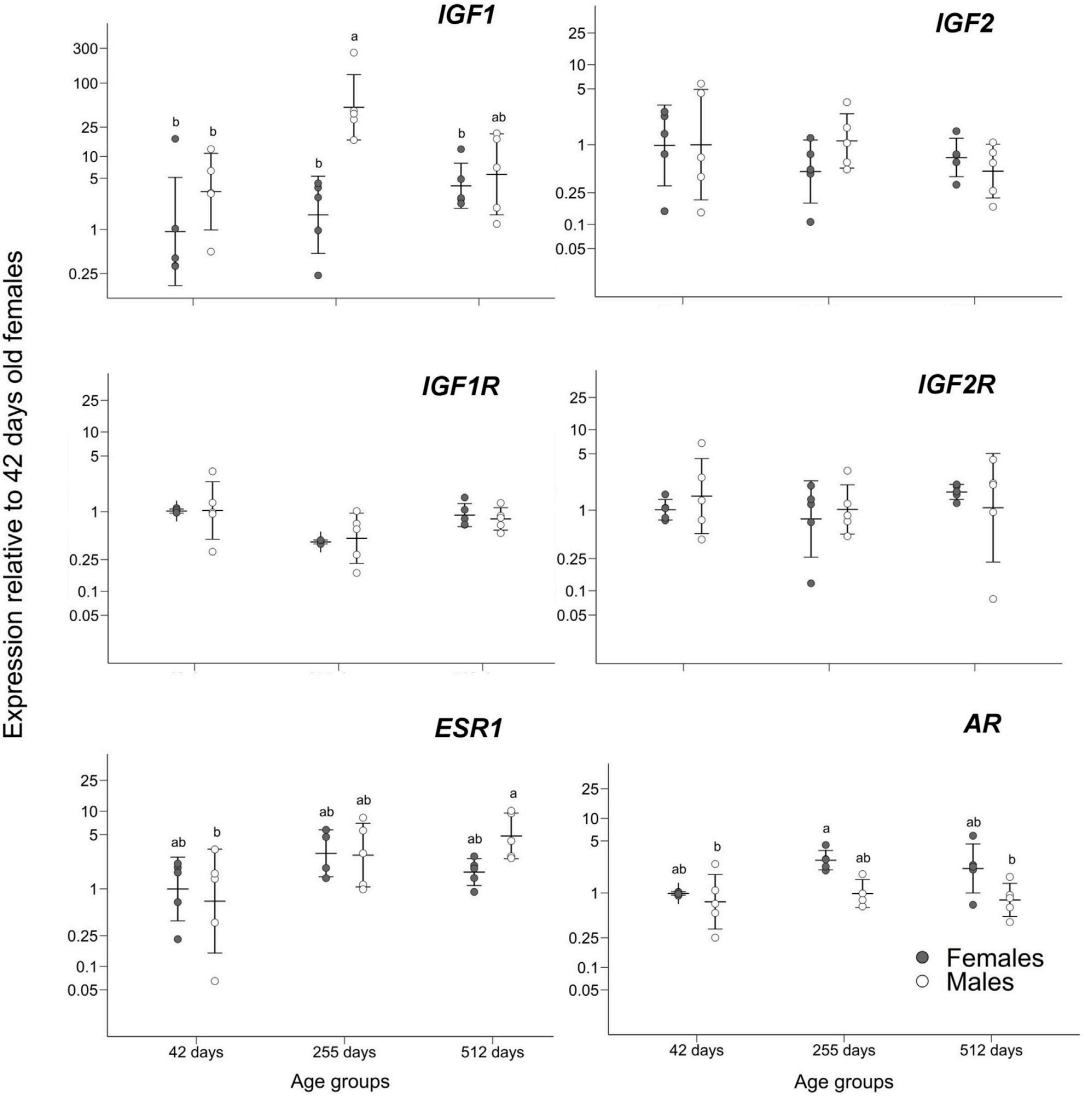
Gene expression relative to the group of 42 days old females. Means ± standard deviations are depicted. Statistically homogenous groups as identified by the *post hoc* Tukey test are depicted where there were significant differences among sex/age groups.

**FIGURE 3:**
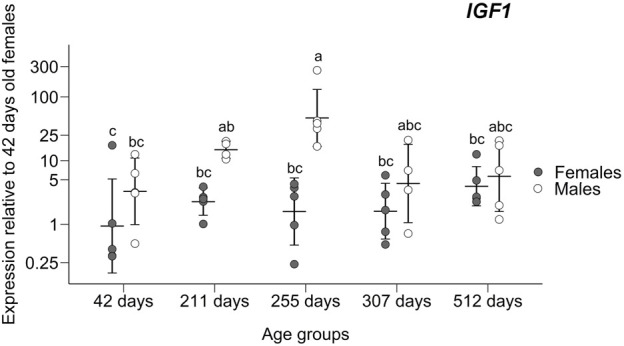
Expression of the insulin-like growth factor-1 gene in livers relative to the group of 42 days old females. Means ± standard deviations are depicted. Statistically homogenous groups identified by the *post hoc* Tukey test are depicted.

A significant difference was found in *IGF1* gene expression in 255 days old males compared to all-female groups and the youngest male group, as pointed by the ANOVA (F_5,24_ = 6.106; *p* = 0.0008) and *post hoc* Tukey tests (significant adjusted *p* < 0.041; non-significant adjusted *p* > 0.106; Figure 2) and prompted us to add two age groups (211 days old and 307 days old individuals) to further verify this trend. After the addition of these four sex/age groups, the overexpression found in 255 days old males was still maintained through ANOVA (F_9,38_ = 5.221; *p* = 0.0001) and *post hoc* Tukey tests (significant adjusted *p* < 0.036; non-significant adjusted *p* > 0.078; Figure 3).

The significant differences in expression for genes *ESR1* and *AR* are not as substantial as for *IGF1*. The ANOVA for *ESR1* test showed a significant difference between age/sex groups (F_5,23_ = 2.882; *p* = 0.036) while the *post hoc* Tukey tests only showed a trend of slightly higher expression in the oldest males compared to the youngest males (adjusted *p* = 0.037; Figure 2) while its expression was comparable among other comparisons (adjusted *p* > 0.128; Figure 2). Also, gene *AR* shows according to ANOVA a significant difference between groups (F_5,22_ = 4.45, *p* = 0.005) but according to the *post hoc* Tukey tests it is only slightly higher expression in 255 days old females compared to the youngest and oldest males (adjusted *p* = 0.018; adjusted *p* = 0.026; Figure 2) while the other *post hoc* comparisons were non-significant (adjusted *p* > 0.083; Figure 2).

Spearman’s correlation coefficient analysis indicated no correlation between ln-transformed *IGF1* gene expression and ln-transformed mean mass of testicles (r = 0.363, *n* = 23, *p* = 0.089) while the correlation between ln-transformed body mass and ln-transformed mean testicle mass was strongly significant (r = 0.887, *n* = 23, *p* <<0.001). Testosterone plasma levels were higher in males than females already well before the separation of male and female growth curves, while estradiol plasma levels were comparable in both sexes before the breakpoint but higher after that in females as described in Kubička et al. (2022) and showed in Supplementary Figures S1, S2. All raw data used in Kubička et al. (2022) are available in the Mendeley database (doi:10.17632/fxcdd6j4sh.1).

## Discussion

We followed the expression of candidate genes that could contribute to sex-specific growth at the stage when SSD develops. Three (*AR, ESR1,* and *IGF1*) out of six monitored genes were found to significantly differ in the expression between the sexes and growth phases. In fact, only one of them (*IGF1*) gave a pattern consistent with its role in sexually dimorphic growth. The overexpression trend in males for gene *IGF1,* especially the noticeable spike in males around the time of departure of male and female growth trajectories, suggests that this gene has a role in SSD development (Figure 3).

As well documented in humans, IGF1 has an important role in bone elongation and growth plate closure (Yakar et al., 2018; Racine and Serrat, 2020). In *P. picta*, the male-specific spike of *IGF1* might delay the senescence of bone growth plates in males, which is translated to male-larger SSD. But what drives the peak in *IGF1* expression in gecko males and why is it missing in females?

In humans, IGF1 levels have been considered to be associated with puberty and occur in both sexes during the so-called pubertal growth spurt (Cole et al., 2015; Juul and Skakkebæk, 2019). A small spike of IGF1 circulating plasma levels was also found in brown house snakes (*Lamprophis fuliginosus*) which could indicate its role in sexual maturity (Sparkman et al., 2010). In *P. picta* the breakpoint and overexpression of *IGF1* in males come a long time after sexual maturity and no correlation between *IGF1* levels and gonad size could be drawn. This further highlights the findings of Kubička et al. (2022) that growth changes in *P. picta* are not associated with sexual maturation and that castration does not alter structural growth in males (Starostová et al., 2013; Kubička et al., 2015). In agreement, circulating levels of testosterone are already sexually dimorphic at the age of 42 days in *P. picta* (Supplementary Figure S2). The size of testicles correlates with male body mass and no significant changes in these parameters coincide with the peak of *IGF1* in males and the onset of sexually dimorphic growth.

As castrated males and ovariectomized females of *P. picta* follow the growth trajectory of control intact males (Starostová et al., 2013; Kubička et al., 2015; Kubička et al., 2017), we expect that the peak of *IGF1* appears without a direct activation by male gonads. *IGF1* expression in livers is controlled by somatotropin (GH) produced by the pituitary gland (e.g., Schwartz and Bronikowski, 2016). GH production in humans increases during childhood and peaks during the pubertal growth spurt (Meinhardt and Ho, 2007). The situation in *P. picta* suggests that it is possible to disconnect the peak of GH*/*IGF1 from sexual maturation during evolution and this observation deserves further study. As stated previously, growth commonly continues in ectotherms after sexual maturation which is not the case for endotherms. This phenomenon is quite interesting and highlights the novelty of the spike in *IGF1* expression we found. This spike of *IGF1* happens after sexual maturation and gives an insight into how in ectotherms growth mechanisms are disconnected from sexual maturation and also how is the ontogeny of SSD detached from reproduction.

Previous growth experiments in *P. picta*, particularly the defeminization of growth by total but not by unilateral ovariectomy and the negative growth effect of exogenous estradiol (Kubička et al., 2017), suggest that the peak in *IGF1* in females of this species can be inhibited by ovarian hormones. This is also consistent with the observation that an increase and a start of cycling of estradiol levels in females roughly coincides with the closure of their bone growth plates, which happens at a smaller body size than in males of *P. picta* (Kubička et al., 2022). We found only a significant but small difference in expression to be higher in the oldest males than in the youngest males in *ESR1* and no differences with the other groups. Also, the pattern of expression found in *ESR1* is not sexually dimorphic as only young and old males differ significantly. We cannot answer why old males have somewhat elevated *ESR1* expression or young males have lower levels compared to each other. Both these groups do not differ in *ESR1* expression from the other experimental groups (Figure 2). However, it does not exclude circulating estrogens as candidates for the modifiers of GH*/*IGF1 expression as the relationship between hormone and receptor levels and their action is not straightforward (Moore and Evans, 1999). Even more importantly, increased female-typical estrogen levels can influence growth via their effect on the GH production/releasing pathway in the brain, which should be tested in the future. Moreover, the estrogen and its receptors can act directly in the growing bones (although this effect would not directly explain the lack of *IGF1* peak in females). In mice, skeletal differences between males and females corresponding with higher mass in males were correlated to IGF1 while both androgens and estrogens were found to have stimulatory effects in males and inhibitory in females, respectively (Callewaert et al., 2010). The role of estrogen receptors in sexual dimorphism has also previously been supported by the report of higher expression of *ESR2* in the cranial skeleton in *Anolis carolinensis*, suggesting its inhibiting role on bone growth (Sanger et al., 2014).

The pattern of *AR* expression through ontogeny in livers does not correspond with the testosterone level difference found between the sexes (Kubička et al., 2022; Supplementary Figure S2). We also know from Kubička et al. (2017) that testosterone increment has no effect on growth in ovariectomized *P. picta* females. The revealed pattern of slightly higher expression of *AR* in 255 days old females compared to the youngest and the oldest males does not seem to be of any biological impact if we also consider that either of these groups shared a similar expression with the remaining groups (Figure 2). Both expression patterns in genes *ESR1* and *AR* seem to be likely not biologically relevant or at the very least not relevant to SSD and could be explained by independent hormonal fluctuations that are not linked to SSD.

*IGF2* did not show any significant differences in the expression between sexes and age cohorts in *P. picta*. The previously assumed divergence in the role of IGF2 as a pre-natal growth factor and IGF1 as a post-natal growth factor has been challenged in reptiles (Schwartz and Bronikowski, 2016; Beatty and Schwartz, 2020) resulting in a necessity to understand their exact role in the ontogeny of growth in vertebrates. If IGF2 has an important role in postnatal growth in *P. picta*, it would be constant throughout development (or at least for the tested age groups) and not associated with SSD.

Future studies should look at the proximate cause of SSD in different lizard groups and test whether the pattern started to be uncovered here for *P. picta* is more general. It would be fascinating to compare ontogeny of growth and *IGF1* expression in monomorphic and female-larger species of the genus *Paroedura* (Starostová et al., 2010). We predict that monomorphic/female-larger species will lack the male-specific peak of *IGF1* expression. A recent study focused on a short-term growth experiment found comparable hepatic expression of *IGF1* in adult males and females in the female-larger iguana *Sceloporus undulatus* (Duncan et al., 2020); however, more systematic studies comparing *IGF1* expression throughout the whole ontogeny from hatching till growth cessation are needed.

## Conclusion

We compared the expression levels of candidate genes and circulating steroid hormones for sex-specific growth modifiers in liver samples collected at different phases of growth in a male-larger gecko species and interpreted the results in the light of previous growth experiments. We found that the ontogeny of sexual dimorphism in this species is likely not connected with male sexual maturation or with plasma circulating levels of male gonadal androgens and expression of *AR,* and *ESR1* in livers. On the other hand, the higher activity of bone growth plates before their closure in males (Kubička et al., 2022) seems to be connected with a significant increase in the expression of *IGF1* at that growth stage. The peak could be inhibited by ovarian hormones produced by active reproductive organs in females.

Genetic variation of the gene *IGF1* played for example a large role in the evolution of body size among dog breeds (Sutter et al., 2007), and variation in *IGF1* is an important mediator of life-history variation among vertebrates (Lodjak and Verhulst, 2020). We demonstrated that the time-specific and sex-specific expression of this gene may play an important role in the development of SSD in reptiles. We hope that future studies monitoring growth patterns and *IGF1* expression across ontogeny in both male-larger and female-larger species will help clear the role of IGF1 in SSD. Further investigation should also uncover if this sex-specific growth mechanism is shared or specific to certain lineages.

## Data Availability

The original contributions presented in the study are included in the article/Supplementary Material, further inquiries can be directed to the corresponding author.

## Supplementary material

Supplemental Figure S1 Variation in estradiol plasma levels among groups. Note that estradiol levels generally increased and started to fluctuate in females after they started reproduction (they were mated around the age of four months). Age groups with measured gene expression are marked.

Supplemental Figure S2 Testosterone plasma levels across sex and age groups. The levels of the androgen became sexually dimorphic quite early in the ontogeny. They stayed low in females and vary a lot but are higher among males. Age groups with measured gene expression are marked.
