# Supplementary figures and images for: Development of male-larger sexual size dimorphism in a lizard: IGF1 peak long after sexual maturity overlaps with pronounced growth in males
Source: Front Physiol. 2022 Aug 10;13:917460. doi: 10.3389/fphys.2022.917460 (PMC9399403; doi:10.3389/fphys.2022.917460)

Supplemental figure 2

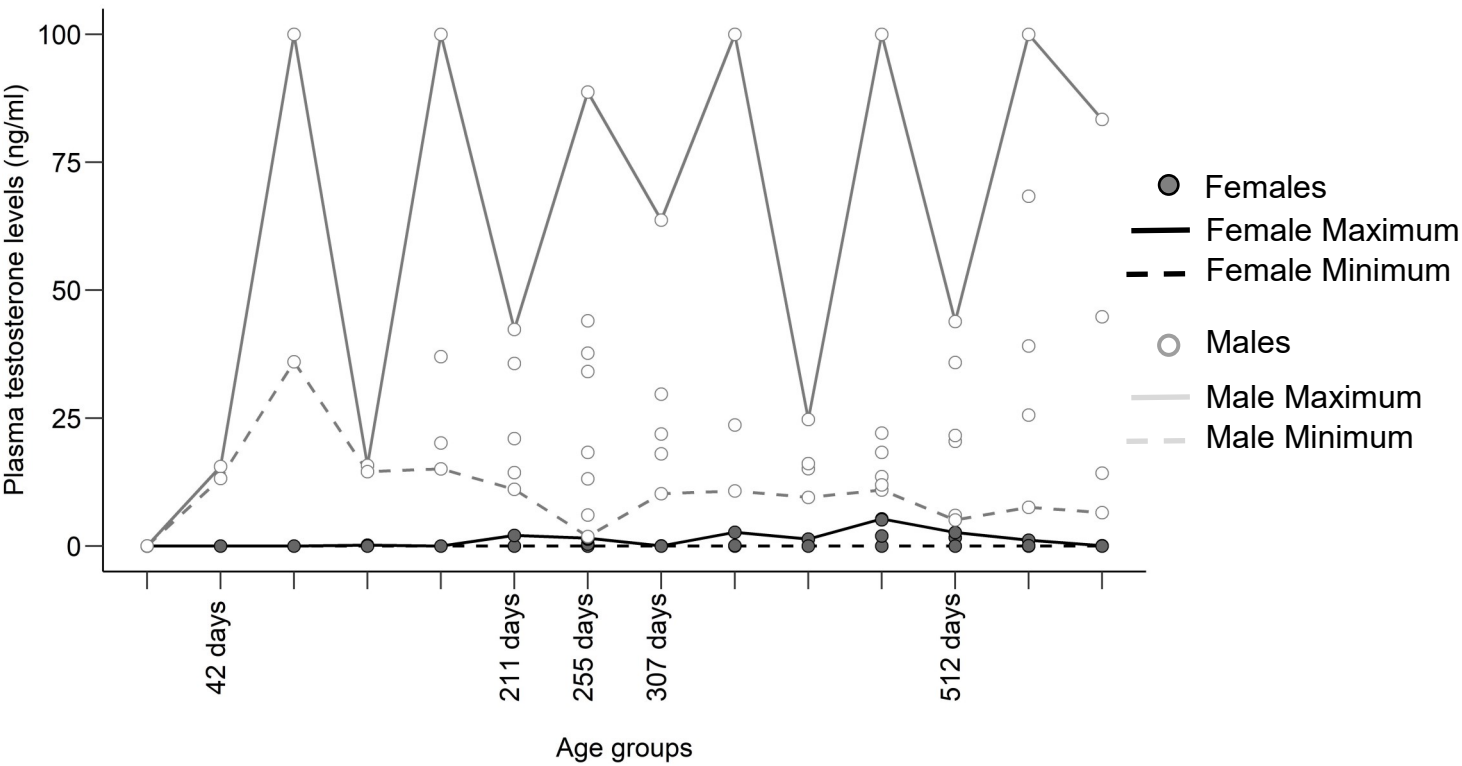

Supplement: Supplementary file 1 [file Image2.pdf]

Supplemental Figure 1

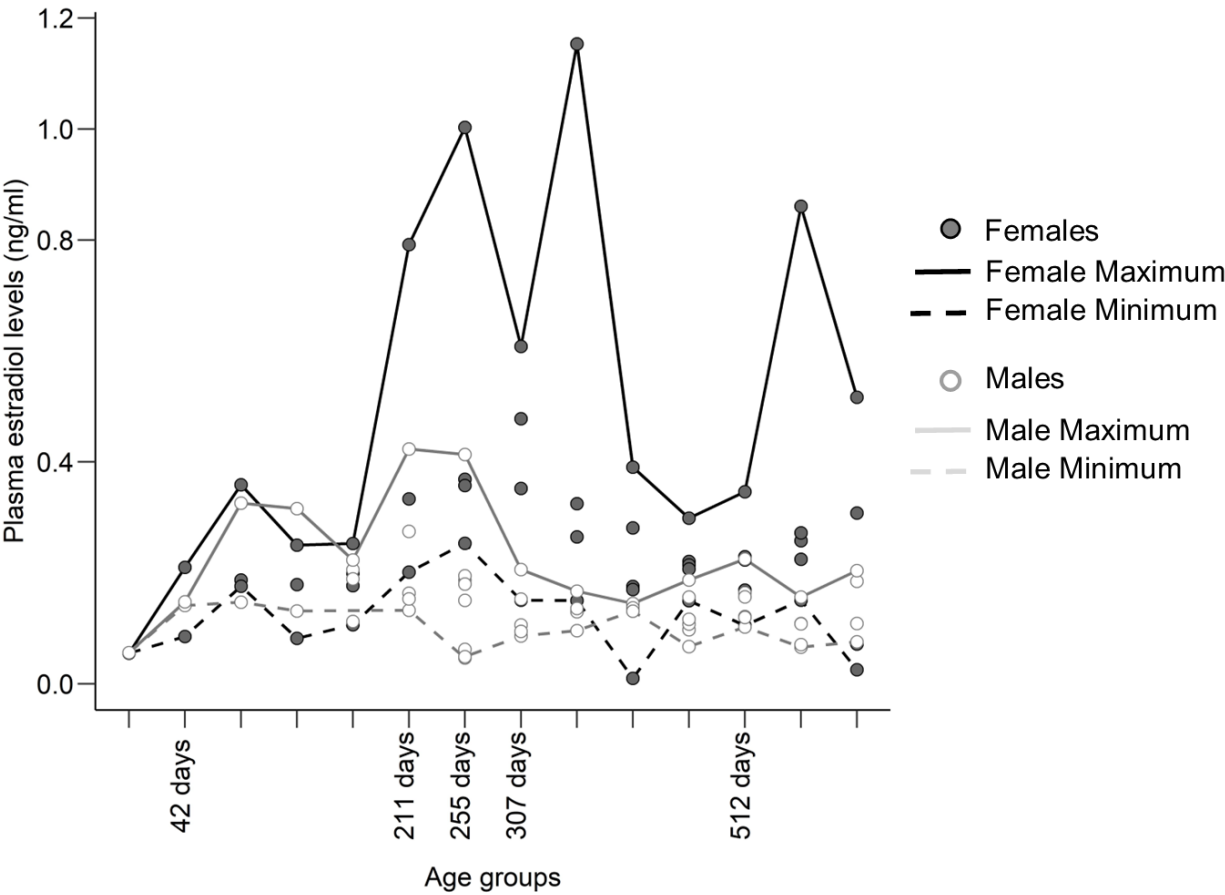

Supplement: Supplementary file 3 [file Image1.pdf]
